# Negative affect instability predicts elevated depressive and generalized anxiety disorder symptoms even when negative affect intensity is controlled for: an ecological momentary assessment study

**DOI:** 10.3389/fpsyg.2024.1371115

**Published:** 2024-04-23

**Authors:** Hedvig Sultson, Carolina Murd, Merle Havik, Kenn Konstabel

**Affiliations:** ^1^Department of Chronic Diseases, National Institute for Health Development, Tallinn, Estonia; ^2^Institute of Psychology, University of Tartu, Tartu, Estonia

**Keywords:** generalized anxiety disorder, emotion dynamics, ecological momentary assessment, depression, affective instability

## Abstract

**Introduction:**

Mood and anxiety disorders are characterized by abnormal levels of positive affect (PA), negative affect (NA) and changes in how emotions unfold over time. To better prevent and treat those disorders, it is crucial to determine which kind of indices of emotion dynamics best predict elevated depressive and generalized anxiety symptoms.

**Methods:**

221 individuals (60 men; mean age = 46 years, SD = 15 years) completed a 7-day ecological momentary assessment study, where their positive and negative affective experience was assessed 5 times a day. For each participant, the intensity, instability, inertia, and differentiation of PA and NA were calculated. The Estonian Emotional State Questionnaire was used to assess depressive and generalized anxiety disorder (GAD) symptoms.

**Results:**

We found that NA and PA intensity, and NA instability predicted elevated depressive and GAD symptoms. Models including NA instability alongside PA and NA intensity showed the best fit for both depression and generalized anxiety, as NA instability alongside other variables significantly increased the odds of having elevated depressive and GAD symptoms. Affective inertia, differentiation, and PA instability were not associated with depressive and GAD symptoms.

**Discussion:**

In addition to the mean levels of affect, it is important to study other emotion dynamic indices such as NA instability, as these offer a more nuanced view of underlying emotion dysregulation processes. This could, in the long-term, help tailor more specific prevention and intervention methods for mood and anxiety disorders.

## 1 Introduction

Emotion dysregulation is considered to be a transdiagnostic risk factor for the development of a wide range of psychiatric disorders, including mood and anxiety disorders (Aldao et al., 2016; Sloan et al., 2017). In fact, high negative affect (NA) in combination with deficient positive affect (PA) is considered to be a core feature of mood and anxiety disorders (Hofmann et al., 2012). Many studies have indeed demonstrated that higher levels of NA and lower levels of PA are characteristic to individuals with depression (Conrad et al., 2008; Mata et al., 2012) and anxiety (Stanton and Watson, 2014).

However, studies using ecological momentary assessment (EMA; Shiffman et al., 2008) indicate that dysregulated affect seen in depression and anxiety does not only manifest in the elevated levels of NA and diminished levels of PA, but can also be seen in how affect unfolds over time (Trull et al., 2015). This area of research is called emotion dynamics and within it, several well-known and validated indices characterizing emotional change have been established (Trull et al., 2015).

Fluctuations in affect, also called affective instability, have perhaps received the most attention in relation to mood and anxiety disorders. Affective instability refers to the moment-to-moment changes in the affect, taking into account both the variability and the temporal dependency of affect ratings (Jahng et al., 2008). Several studies have shown greater NA instability in depressive (Thompson et al., 2012; Schoevers et al., 2021) and anxiety disorders (Pfaltz et al., 2010; Schoevers et al., 2021). Some studies (e.g., Schoevers et al., 2021) have also shown greater PA instability in depression and anxiety disorders, whereas others (Peeters et al., 2006; Thompson et al., 2012) have not found any differences in comparison to individuals with no psychopathology.

Another widely used index of emotion dynamics is affective inertia. Affective inertia reflects the extent to which individuals’ affective states persist from one moment to the next, essentially reflecting the resistance to change (Kuppens et al., 2010). Affective inertia is captured as the within-person autocorrelation of affect measured repeatedly over time (Jahng et al., 2008; Trull et al., 2015). Greater affective inertia has often been associated with depression (Kuppens et al., 2010; Koval et al., 2013), such as that higher affective inertia in both PA and NA have been shown to prospectively predict future onset of major depressive disorder (MDD) (Kuppens et al., 2012). Therefore, some researchers have proposed that higher affective inertia could serve as an early risk factor for depression (Kuppens et al., 2012; van de Leemput et al., 2014), whereas others argue that high inertia of affect might be a consequence of depression (Houben and Kuppens, 2020; Minaeva et al., 2021).

Lastly, emotional differentiation, also known as emotional granularity (Barrett, 2004), is also considered to be an important indicator of emotion dynamics. Emotional differentiation refers to the extent to which the individual can differentiate between the emotions of the same valence (Barrett et al., 2001). In other words, individuals with high emotional differentiation are able to describe their feelings in a more nuanced way and are therefore able to be more flexible in choosing the most suitable emotion regulation strategy (Trull et al., 2015). Given that mood and anxiety disorders are associated with difficulties in emotion regulation that often result in dysregulated NA and PA, it is likely that individuals with depression and generalized anxiety disorder (GAD) symptoms also show lower level of (negative) emotional differentiation. In support of this, Demiralp et al. (2012) found that patients with MDD had significantly lower NA differentiation compared to healthy participants, whereas no differences in PA differentiation between the groups were found. Similar results were also found in a non-clinical sample of students (Erbas et al., 2014), where lower NA differentiation was associated with elevated depressive symptoms.

All in all, it is important to investigate subtle changes in affect, as these help more precisely elucidate the underlying mechanisms of mental disorders. For example, Bosley et al. (2019) found that instability of NA was uniquely associated with the severity and the treatment response of GAD symptoms, whereas no specific associations were found for depression. Better understanding of such underlying mechanisms can help design more precise, targeted and/or personalized prevention and intervention strategies.

Nonetheless, Dejonckheere et al. (2019) argue that statistically, complex emotion dynamic indices add little additional explanatory value in predicting psychological well-being when investigated alongside the mean level of PA and NA. Thus, besides looking at how individual predictors are associated with psychopathology, it is important to investigate whether the emotional dynamic measure adds anything to the statistical prediction of well-being above and beyond the mean level of NA and PA (Dejonckheere et al., 2019).

Therefore, in this study, we had two main aims. First, we investigated how each index of emotion dynamics predicts^1^ elevated depressive and generalized anxiety disorder symptoms. As the significant associations between each emotion dynamic index and symptomatology have been more studied in the context of depression, and less so in the context of anxiety disorders, the hypotheses for individual predictors were the following: (a) the intensity of NA and PA predict depressive and GAD symptoms, (b) inertia of PA and NA predict depressive symptoms, (c) NA instability predicts depressive and GAD symptoms, (d) NA differentiation predicts depressive symptoms. Secondly, we explored which set of emotion dynamic indices best predict elevated depressive and generalized anxiety symptoms, and whether these predictors are superior to the mean level of PA and NA. No *a priori* hypotheses were formed for the best set of predictors.

## 2 Materials and methods

### 2.1 Sample characteristics

The sample for this study was derived from the Estonian National Mental Health Study (EMHS; Laidra et al., 2023), conducted in 2021–2022. Current sample consisted of 221 individuals (60 men, 161 women) with the mean age of 46 years (SD = 15 years) who filled out the Wave 2 survey of EMHS and simultaneously participated in the ecological momentary assessment-based validation study.

Out of 221 participants, 51.4% had higher education, 45.4% secondary education or vocational education, and 3.2% primary education. 30.9% of participants had a self-reported net income of >1,400 EUR, 37.3% 851–1,400 EUR, 19.1% 451–850 EUR, and 12.7% up to 450 EUR. The study design was approved by the Research Ethics Committee of the National Institute for Health Development, Estonia.

### 2.2 Measures

#### 2.2.1 Depressive and generalized anxiety disorder symptoms

The modified version of the Estonian Emotional State Questionnaire (EST-Q; Aluoja et al., 1999), EST-Q2 (Ööpik et al., 2006), was used to measure depressive and generalized anxiety disorder symptoms. EST-Q2 is a 28-item self-report measure used to screen for depressive and anxiety symptoms in population-based studies (Aluoja et al., 2004; Stickley and Leinsalu, 2018; Laidra et al., 2023) and among primary care patients (Ööpik et al., 2006). Participants were instructed to rate on a 5-point scale ranging from 0 to 4 (0 = not at all… 4 = all the time) the extent to which each problem has troubled him/her during the last 4 weeks.

EST-Q2 has previously been validated on a sample of inpatients with depressive and anxiety disorders, as well as on a population sample (Aluoja et al., 1999). Depression, Anxiety and Agoraphobia-Panic subscales have been shown to reliably distinguish patient groups (i.e., depressive disorder, generalized anxiety disorder, agoraphobia with panic disorder, respectively) from each other and are therefore considered to be discriminative across diagnostic categories according to DSM-IV and ICD-10 diagnoses (Aluoja et al., 1999).

Originally, EST-Q2 consists of 6 subscales: Depression, Anxiety, Agoraphobia-Panic, Social anxiety, Fatigue, and Insomnia. In the analyses, we only used EST-Q2 Depression and Anxiety subscales, as these represent the cognitive-affective components of depression and generalized anxiety disorder and are therefore suitable for our research questions. Panic-Agoraphobia subscale was omitted, as it represents a more limited set of anxiety symptoms (i.e., panic disorder and agoraphobia).

EST-Q2 Depression subscale (*α* = 0.92) consists of 8 items measuring the cognitive and affective symptoms of depression, such as feelings of sadness, loneliness, and worthlessness, hopelessness about the future, loss of interest and inability to feel joy, self-accusations, and recurrent thoughts of death and suicide (Aluoja et al., 1999). The cut-off score > 11 is used to identify people with elevated depressive symptoms, as previous studies have shown that this cut-off score correctly identifies 81.5% of patients with the ICD-10 clinical diagnosis of a depressive episode (Ööpik et al., 2006).

EST-Q2 Anxiety subscale (*α* = 0.88) consists of 6 items reflecting the cognitive and affective aspects of generalized anxiety disorder, such as excessive worry about many different things, being easily startled, feeling anxious or frightened, inability to relax, feeling restless, and being easily irritated (Aluoja et al., 1999). The cut-off score > 11 is used to identify people with elevated generalized anxiety symptoms (Ööpik et al., 2006).

#### 2.2.2 Ecological momentary assessment data

In the validation study, ecological momentary assessment (EMA; Shiffman et al., 2008) was used to acquire data on momentary positive and negative affect. In the EMA study, participants were prompted 5 times a day (from 9 am to 9 pm), for 7 consecutive days, to answer a short questionnaire pertaining to the intensity of their emotional experience. Specifically, participants had to rate on a 7-point scale (0 – ‘not at all’ … 6 – ‘very strongly’) on how strongly they were currently feeling the following emotions: joy/excitement, satisfaction/relaxation, worry/anxiety, sadness/disappointment, irritation/anger, tension/stress, tiredness/listlessness. Two items per emotion rating were added to reduce participant’s burden and to concisely capture a wider affective experience of a participant. For each day, prompts were timed randomly within five 90-min time slots (9.00–10.30 am, 11.30–1.00 pm, 2.00–3.30 pm, 4.30–6.00 pm, 7.00–8.30 pm), and each prompt was open for an hour.

Additionally, participants had to answer questions about emotion regulation strategies, sleep, physical activity, and alcohol consumption. For this article, only data on the momentary emotional experience is used, and the full version of the EMA questionnaire can be found in the Supplement.

### 2.3 Procedure

A more detailed account on the recruitment as well as the methods used can be found in Laidra et al. (2023), but in short, EMHS was a methodologically complex population-based study that combined repeated longitudinal and cross-sectional surveys with registry-linked data and additional smaller studies. As the aim of the study was to provide a comprehensive overview of mental health problems and its correlates in the Estonian population, regionally representative stratified sampling was used to recruit 20,000 permanent residents of Estonia into the study.

**Figure 1:**
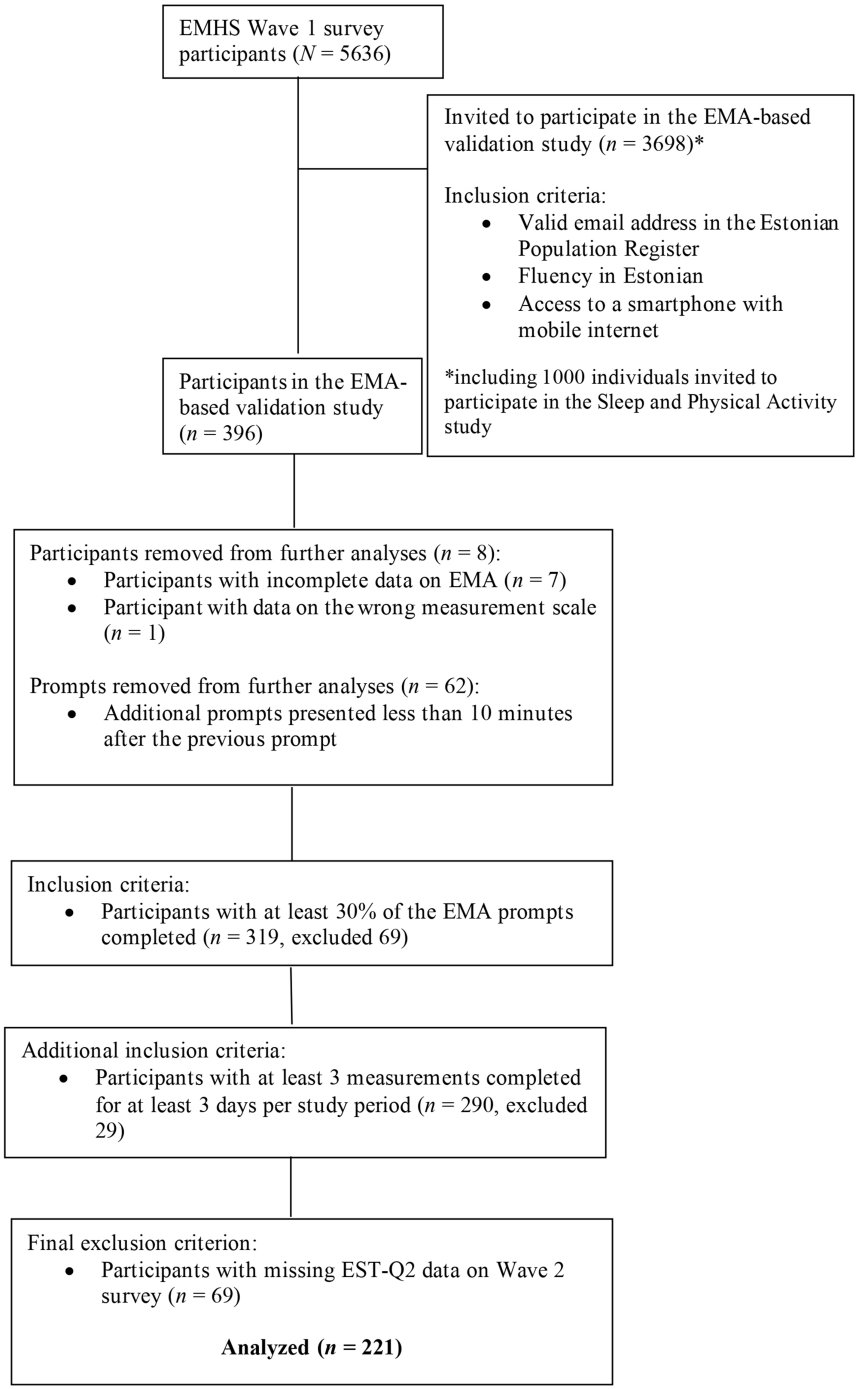
Participant flow of the EMA-based validation study of EMHS.

The survey part of EMHS consisted of three data collection waves that were conducted between January 2021 and February 2022. Important for this article, Wave 1 respondents who had filled out the survey in Estonian were invited to participate in the EMA-based validation study that was carried out in parallel to the Wave 2 survey in May–June 2021. In total, 3,698 respondents fluent in Estonian and with a valid email address in the Estonian Population Register were invited to participate in the validation study. Out of these 3,698 participants, 1,000 individuals who lived close to the study center area were additionally asked to wear an activity monitor for the duration of the validation study and give 4 saliva samples for cortisol assessment (Sleep and Physical activity subsample). More details on the EMHS can be found in Laidra et al. (2023). Participant flow of the EMA-based validation study of EMHS is also depicted in Figure 1.

The EMA part of the validation study, following the completion of the Wave 2 survey, was carried out using the open source survey framework formr^2^ (Arslan et al., 2020). Participants chosen for the validation study received a personalized link at the end of the Wave 2 survey or via email, directing them to formr. After confirming their interest in taking part in the validation study, participants were asked to provide a mobile phone number, to which they were sent instant messages containing the link to the EMA questionnaire. Instant messages were sent via the Direct Messenger SMS API.^3^

### 2.4 Data selection

The response rate for the EMA study was 64.6%. In total, there were 8,706 measurements (*N* = 389) after removing prompts with incomplete data (*n* = 808). Due to technical problems, additional 88 erroneous prompts were removed from the dataset [i.e., data on the wrong measurement scale across the ratings of one participant (*n* = 26), and additional prompts that were presented less than 10 min after the correct prompt (*n* = 62)]. One participant completed the EMA questionnaire twice and therefore, only data upon the first completion was retained (*n* = 23 prompts removed).

For the purpose of this study, only participants who had successfully completed at least 30% of the prompts (i.e., at least 10 prompts out of 35) were included in the analyses. Additional inclusion criteria were also applied, so that only days that included at least 3 answered prompts per participant were included, and subsequently, only participants with at least 3 prompts on at least 3 days were retained. Lastly, after excluding participants with missing EST-Q2 data in the Wave 2, the final sample consisted of 221 participants (1,391 days, 5,880 measurements).

In comparison to the EMHS Wave 2 survey (*N* = 3,760), the final sample for EMA included less males (27% vs. 38% in the survey sample), younger individuals (mean age = 46 vs. mean age = 57 in the survey sample), and higher prevalence of elevated generalized anxiety (23.6% vs. 17.3% in the survey sample) and depressive (28.3% vs. 22.4% in the survey sample) symptoms.

### 2.5 Data analyses

Prior to the data analyses, emotion dynamic indices were calculated for each participant.

To quantify positive and negative affect intensity, mean levels of PA and NA were calculated by taking the average of the items measuring PA (2 items: joy/excitement, satisfaction/relaxation; *α* = 0.9) and NA (4 items: worry/anxiety, sadness/disappointment, irritation/anger, tension/stress; *α* = 0.91), respectively. These indices of PA and NA intensity were calculated for each day, and then averaged across the 7-day study period (for each participant).

For positive and negative affect instability, root mean squared successive differences (RMSSD; Jahng et al., 2008) of the mean PA and NA were calculated for each day, and then averaged across the 7-day study period (for each participant).

For positive and negative affect inertia, autocorrelations of PA and NA ratings were calculated for each individual. Prior to the calculation, missing values were inserted after the last observation of each day, so that the calculated autocorrelation for PA and NA would capture the average autocorrelation across the 7-days for each individual. For further analyses, PA and NA inertia indices were Fisher’s z-transformed. Functions ‘rmssd’ and ‘autoR’ from the R package psych (Revelle, 2023) were used for the calculation of PA/NA instability and inertia, respectively.

For positive and negative affect differentiation, average Fisher’s z-transformed Pearson’s correlations were calculated for each participant. As only two items measured PA, PA differentiation was captured by the Pearson’s correlation coefficient (calculated for each participant) that was Fisher’s z-transformed for subsequent analyses. For NA differentiation, the average of the Fisher’s z-transformed Pearson’s correlation coefficients across all items measuring NA were calculated for each participant. Higher values indicate poorer emotional differentiation (Barrett et al., 2001).

Data preparation and all subsequent data analyses were conducted in the statistical computing R environment 4.2.1 (R Core Team, 2021). To control for the family-wise error rate due to multiple testing, significance threshold was set to 0.01, and 99% confidence intervals were used in all analyses. As depressive and GAD symptoms were found to be more prevalent among women and in younger adults (Laidra et al., 2023), age and gender were added as covariates in all models.

To compare participants with elevated depressive and generalized anxiety symptoms to those without, t-tests were conducted across all study variables pertaining to emotion dynamics. Next, individual logistic regression analyses were carried out for each emotion dynamic index. Specifically, binary logistic regression analyses were conducted with elevated depressive symptoms (coded as 0 – normal, 1 – elevated) or elevated generalized anxiety symptoms (coded as 0 – normal, 1 – elevated) as the binary dependent variable, and the emotion dynamic index as the predictor variable. All individual predictor variables were standardized before conducting the analyses. Age and gender were added as covariates in all analyses, and odds ratios with 99% CI were computed using the R package Epi (Carstensen et al., 2022).

To investigate whether mean levels of PA and NA predict elevated depressive and GAD symptoms (initial models), two binary logistic regression models were conducted with the EST-Q2 depressive or generalized anxiety symptoms as the binary dependent variable, and PA and NA intensity as continuous predictor variables, standardized prior to the analyses. In both models, age and gender were added as covariates.

Finally, to test whether adding more specific indices of emotion dynamics into initial models improve model fit, only statistically significant predictors (i.e., NA instability) from the individual logistic regression analyses were added to the initial models. PA and NA intensity were also included in the final models, as it is recommended to control for the mean level of affect [i.e., the measures of emotional intensity and instability are not mathematically independent from each other (see Koval et al., 2013)], and therefore interactions between them are often seen (Ebner-Priemer et al., 2009). To test whether adding another predictor into the model improves model fit, ‘anova’ function in R was used. AIC criteria and log-likelihood-based pseudo-R^2^ values were used to determine the best models.

In parallel, all of the abovementioned models were tested in a linear regression analysis [using the R package lm.beta package for standardized regression coefficients (Behrendt, 2023)] with the continous score of Depression or Anxiety on the EST-Q2 subscale as the dependent variable. As the results of linear regression models were similar to logistic regression models, these models will be added as a Supplementary material.

R code detailing the calculation of emotion dynamic indices (code 1) and subsequent data analyses (code 2) has been uploaded.^4^ Due to confidentiality restrictions, full dataset of this article cannot be made publicly available, but a synthetic dataset [using R package synthpop (Nowok et al., 2016)] has been made to try out the models (code 2) used in this article. The synthetic dataset in randomly generated to match the covariation structure in the actual data, and thus, will produce similar (but not identical) results reported in this article.

## 3 Results

### 3.1 Descriptive data

According to the EST-Q2 Depression and Anxiety subsales, 28% of the sample had elevated depressive symptoms, and 23.7% elevated GAD symptoms. Out of 69 participants who showed elevated levels of depressive and GAD symptoms, 62.3% demonstrated elevated levels of both depressive and generalized anxiety symptoms (*n* = 43), 11.6% (*n* = 8) elevated GAD symptoms only, and 26% (*n* = 18) elevated depressive symptoms only. More information on the descriptive statistics of the sample and on the differences in the emotion dynamic indices can be found in Table 1 and Table 2, respectively.

**Table 1:** Descriptive statistics of the sample.

	Depressive symptoms		Generalized anxiety symptoms	
	Below the cut-off (normal)	Above the cut-off (elevated)	Below the cut-off (normal)	Above the cut-off (elevated)
*n* (%)	157 (72.0)	61 (28.0)	167 (76.3)	52 (23.7)
Mean age (SD)	48 (14)	38 (14)	47 (15)	39 (14)
	*n* (%)	*n* (%)	*n* (%)	*n* (%)
Women	109 (69.0)	49 (31.0)	118 (73.7)	42 (26.3)
Men	48 (80.0)	12 (20.0)	49 (83.1)	10 (16.9)

SD, standard deviation.

**Table 2:** Descriptive data for the intensity, instability, inertia, and differentiation of negative and positive affect.

	Depressive symptoms				Generalized anxiety symptoms			
	Below the cut-off (normal)	Above the cut-off (elevated)			Below the cut-off (normal)	Above the cut-off (elevated)		
Variable	M (SD)	M (SD)	*t*	*p*	M (SD)	M (SD)	*t*	*p*
PA intensity	2.84 (1.20)	2.07 (0.88)	5.22	**< 0.001**	2.80 (1.19)	2.02 (0.81)	5.29	**< 0.001**
NA intensity	0.58 (0.60)	1.53 (0.97)	−7.13	**< 0.001**	0.57 (0.57)	1.72 (0.96)	−8.20	**< 0.001**
PA instability	1.0 (0.39)	1.18 (0.53)	−2.49	0.0149	1.0 (0.39)	1.21 (0.56)	−2.40	0.0193
NA instability	0.51 (0.30)	0.88 (0.41)	−6.42	**< 0.001**	0.51 (0.29)	0.93 (0.43)	−6.50	**< 0.001**
PA inertia	0.40 (0.42)	0.32 (0.36)	1.38	0.1711	0.38 (0.40)	0.33 (0.42)	0.81	0.4213
NA inertia	0.27 (0.43)	0.31 (0.40)	−0.54	0.5895	0.27 (0.43)	0.30 (0.38)	−0.42	0.6761
PA differentiation	0.66 (0.51)	0.62 (0.49)	0.51	0.6079	0.64 (0.50)	0.70 (0.53)	−0.71	0.4825
NA differentiation	0.38 (0.33)	0.48 (0.32)	−2.09	0.0387	0.38 (0.33)	0.51 (0.31)	−2.43	0.0169

NA, negative affect; PA, positive affect; SD, standard deviation.

### 3.2 Individual predictors

**Figure 2:**
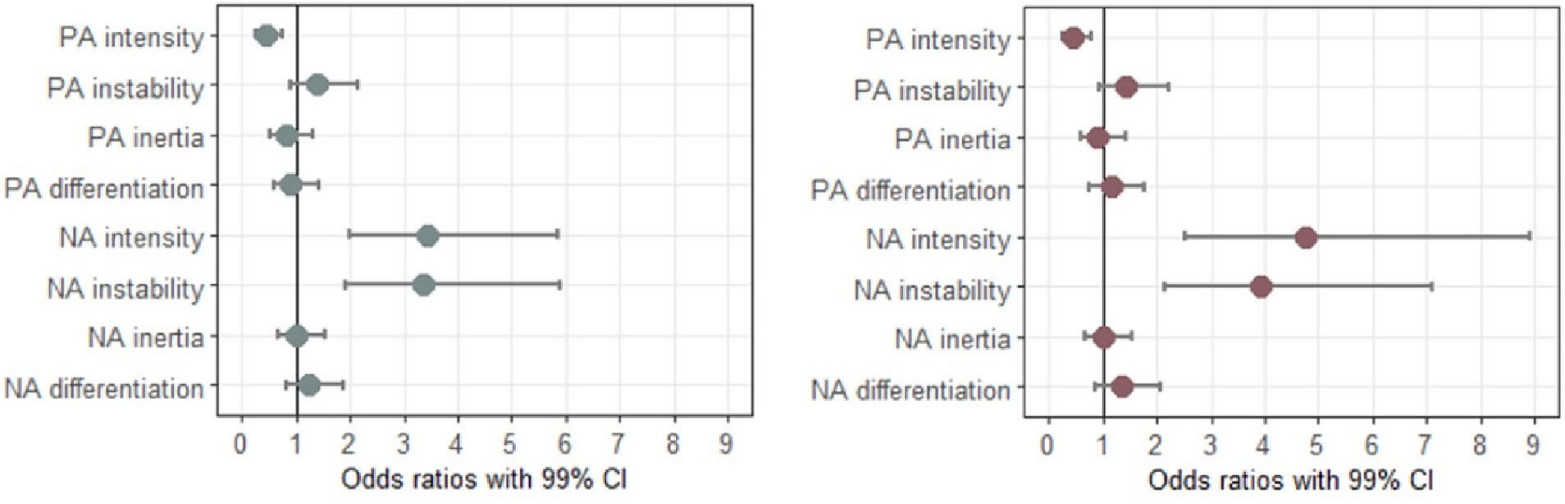
Odds ratios with 99% CI for individual emotion dynamic indices predicting elevated depressive (left panel) and generalized anxiety (right panel) symptoms. All odds ratios were adjusted for gender and age, and all predictor variables were standardized prior to the analyses.

The results pertaining to how each emotion dynamic index predicts elevated depressive and generalized anxiety symptoms are presented in Table 3 and illustrated in Figure 2.

**Table 3:** Odds ratios for individual emotion dynamic indices predicting elevated depressive and generalized anxiety symptoms.

	Elevated depressive symptoms			Elevated generalized anxiety symptoms		
Variable	OR	99% CI	*p*	OR	99% CI	*p*
PA intensity	0.45	0.27–0.76	**< 0.001**	0.45	0.26–0.77	**< 0.001**
NA intensity	3.42	1.99–5.87	**< 0.001**	4.74	2.52–8.91	**< 0.001**
PA instability	1.40	0.92–2.14	0.0387	1.44	0.94–2.23	0.0289
NA instability	3.37	1.93–5.89	**< 0.001**	3.92	2.16–7.10	**< 0.001**
PA inertia	0.84	0.54–1.31	0.316	0.92	0.59–1.43	0.6110
NA inertia	1.01	0.67–1.54	0.932	1.01	0.66–1.56	0.9356
PA differentiation	0.92	0.59–1.42	0.6032	1.15	0.75–1.76	0.4096
NA differentiation	1.25	0.82–1.90	0.1775	1.36	0.88–2.09	0.0684

CI, confidence interval; NA, negative affect; OR, odds ratio, PA, positive affect. All predictors were adjusted for age and gender, and standardized prior to the analyses.

Out of all indices, only PA intensity, NA intensity, and NA instability significantly predicted elevated depressive and GAD symptoms. Specifically, the odds of having elevated depressive and generalized anxiety symptoms were significantly increased when NA intensity (OR = 3.42, 99% CI 1.99–5.87 for depression, OR = 4.74, 99% CI 2.52–8.91 for generalized anxiety) and NA instability (OR = 3.37, 99% CI 1.93–5.89 for depression, OR = 3.92, 99% CI 2.16–7.10 for generalized anxiety) increased. Likewise, the odds of having elevated depressive and generalized anxiety symptoms were significantly decreased when PA intensity increased (OR = 0.45, 99% CI 0.27–0.76 for depression, OR = 0.45, 99% CI 0.26–0.77 for generalized anxiety). In contrast to NA instability, PA instability did not significantly predict the odds of having elevated depressive or GAD symptoms.

### 3.3 Models

In both initial models (see Table 4), NA intensity significantly predicted elevated levels of depressive and generalized anxiety symptoms. Specifically, for every SD increase in NA intensity, the odds of having elevated depressive and GAD symptoms were increased at least 3 times (OR = 3.01, 99% CI 1.75–5.18 for depression, OR = 4.21, 99% CI 2.23–7.94 for generalized anxiety). However, with NA intensity included in the models, PA intensity did not significantly predict elevated depressive and GAD symptoms.

**Table 4:** Results of the initial and final logistic regression models predicting elevated depressive and generalized anxiety disorder symptoms.

	Elevated depressive symptoms (*n* = 218)											
	Initial model						Final model					
Variable	*B*	SE	*Z*	*p*	OR	99% CI	*B*	SE	*Z*	*p*	OR	99% CI
(Intercept)	0.36	0.93	0.39	0.6961	1.44	0.13–15.65	0.16	0.95	0.17	0.8664	1.17	0.10–13.66
Gender (female)	0.36	0.43	0.82	0.4097	1.43	0.47–4.36	0.47	0.45	1.05	0.2953	1.60	0.51–5.04
Age	−0.05	0.01	−3.77	**< 0.001**	**0.95**	**0.92–0.98**	−0.05	0.01	−3.79	**< 0.001**	**0.95**	**0.91–0.98**
PA intensity	−0.58	0.24	−2.43	0.0151	0.56	0.30–1.03	−0.67	0.25	−2.68	**0.0075**	**0.51**	**0.27–0.98**
NA intensity	1.10	0.21	5.25	**< 0.001**	**3.01**	**1.75–5.18**	0.66	0.25	2.66	**0.0078**	**1.94**	**1.02–3.68**
NA instability							0.77	0.27	2.85	**0.0044**	**2.15**	**1.08–4.30**
	Elevated generalized anxiety disorder symptoms (*n* = 219)											
	Initial model						Final model					
Variable	*B*	SE	*Z*	*p*	OR	99% CI	*B*	SE	*Z*	*p*	OR	99% CI
(Intercept)	−0.14	1.03	−0.14	0.8897	0.87	0.06–12.17	−0.37	1.06	−0.35	0.7293	0.69	0.05–10.65
Gender (female)	0.18	0.47	0.38	0.7013	1.20	0.35–4.06	0.27	0.49	0.55	0.5841	1.31	0.37–4.58
Age	−0.04	0.01	−2.87	**0.0041**	**0.96**	**0.92–1.0**	−0.05	0.02	−2.90	**0.0037**	**0.96**	**0.92–0.99**
PA intensity	−0.55	0.27	−1.99	0.0463	0.58	0.29–1.17	−0.65	0.29	−2.24	0.0249	0.52	0.25–1.10
NA intensity	1.44	0.25	5.84	**<0.001**	**4.21**	**2.23–7.94**	1.02	0.28	3.63	**<0.001**	**2.77**	**1.34–5.70**
NA instability							0.77	0.28	2.73	**0.0064**	**2.16**	**1.04–4.48**

B, regression coefficient; CI, confidence interval; NA, negative affect; OR, odds ratio; PA, positive affect; SE, standard error. In all models, PA intensity, NA intensity (and NA instability) were standardized prior to the analyses.

When comparing initial and final models predicting elevated depressive and generalized anxiety symptoms, final models with an added predictor of NA instability fit the data better. Final models were significantly different from initial models (*χ*^2^ = 8.75, *p* = 0.0031 for depressive symptoms, *χ*^2^ = 7.86, *p* = 0.0051 for generalized anxiety symptoms) and had lower AIC (AIC = 181.25 for depressive symptoms, AIC = 156.69 for generalized anxiety symptoms) compared to the initial models (AIC = 188 for depressive symptoms, AIC = 162.55 for generalized anxiety symptoms). Pseudo-*R*^2^ was also higher for both final models (adjusted Pseudo-*R*^2^ = 0.45 for depressive symptoms, adjusted Pseudo-*R*^2^ = 0.56 for generalized anxiety symptoms) compared to the initial models (adjusted Pseudo-*R*^2^ = 0.41 for depressive symptoms, adjusted Pseudo-*R*^2^ = 0.53 for generalized anxiety symptoms).

Thus, in both final models (see Table 4), NA instability significantly predicted elevated symptoms of depression and generalized anxiety even when NA intensity was controlled for. Specifically, for every SD increase in NA instability, the odds of having elevated depressive and GAD symptoms were increased at least 2.1 times (OR = 2.15, 99% CI 1.08–4.30 for depression, OR = 2.16, 99% CI 1.04–4.48 for generalized anxiety).

The odds of having elevated GAD symptoms were also significantly increased when NA intensity increased (OR = 2.77, 99% CI 1.34–5.70), whereas the role of PA intensity was not significant. Similarly, the odds of having elevated depressive symptoms were significantly increased when NA intensity increased (OR = 1.94, 99% CI 1.02–3.68). In contrast to generalized anxiety symptoms, the odds of having elevated depressive symptoms were significantly decreased when PA intensity increased (OR = 0.51, 99% CI 0.27–0.98).

However, in the final linear regression model for depressive symptoms (see Supplementary Table S3), only NA intensity significantly predicted depressive symptoms (*B* = 0.47, SE = 0.58, *t* = 6.67, *p* < 0.001), whereas PA intensity (*B* = −0.13, SE = 0.31, *t* = −2.47, *p* = 0.0145) and NA instability (*B* = 0.13, SE = 1.26, *t* = 1.88, *p* = 0.0617) did not. In contrast, intensity of PA (*B* = −0.15, SE = 0.25, *t* = −2.75, *p* = 0.0065) and NA (*B* = 0.33, SE = 0.45, *t* = 4.69, *p* < 0.001), and NA instability (*B* = 0.27, SE = 0.98, *t* = 4.03, *p* < 0.001) were all significant predictors of GAD symptoms in the final linear regression model.

## 4 Discussion

In this study, we explored how different indices of emotion dynamics are associated with elevated depressive and generalized anxiety symptoms in a population-based sample. Specifically, we used ecological momentary assessment over a period of 7 days and looked at the instability, inertia and differentiation of positive and negative affect. We also tested which set of emotion dynamic indices best predict elevated depressive and generalized anxiety disorder symptoms, and further, whether the effect of those predictors exceeds the effect of PA/NA intensity.

### 4.1 Individual predictors

We hypothesized that lower levels of PA and higher levels of NA are associated with elevated depressive and generalized anxiety symptoms, and this hypothesis was supported. We found that lower level of PA intensity and higher level of NA intensity increased the odds of having elevated depressive and generalized anxiety symptoms. These results are in accordance with numerous studies that have linked depression and generalized anxiety to elevated NA and diminished PA (Conrad et al., 2008; Mata et al., 2012; Stanton and Watson, 2014).

In line with the second hypothesis, elevated depressive and generalized anxiety symptoms were both associated with higher NA instability. This is in accordance with the literature wherein greater instability of NA has been shown to characterize depressive (Thompson et al., 2012; Schoevers et al., 2021) and anxiety disorders (Pfaltz et al., 2010; Schoevers et al., 2021). Specifically, greater NA instability might indicate both a higher sensitivity or reactivity to the environment, resulting in higher fluctuations in affect (Trull et al., 2015), and a higher use of maladaptive emotion regulation strategies (Gratz and Tull, 2010) that are unsuccessful in downregulating NA. Thus, future studies should look into more specific emotion regulation strategies contributing to higher NA fluctuations, and consequently investigate, whether these strategies differ for depressive and anxiety disorders.

Contrary to our expectations, affective inertia (PA or NA) was not associated with elevated depressive symptoms. This is surprising, as there is ample evidence linking higher inertia of PA and NA to depressive symptoms (e.g., Kuppens et al., 2010; Brose et al., 2015; Koval et al., 2016) and depression severity (Koval et al., 2012), thereby rendering it an important vulnerability factor for depression. Similarly, NA differentiation did not predict elevated depressive symptoms, which is in contrast to the studies (Demiralp et al., 2012; Erbas et al., 2014) demonstrating that less differentiated negative emotions are characteristic to individuals with depression.

To explain these null findings pertaining to emotion differentiation and inertia, some nuances of the study design and sample should be considered. Perhaps no effect of emotional inertia was found, as we investigated a population-based sample of individuals, instead of comparing clinical and non-clinical groups. As some studies argue that emotional inertia might be the consequence of depression (Houben and Kuppens, 2020; Minaeva et al., 2021), the association between inertia of affect and depression might be more evident when studying clinical population. This explanation is insufficient, however, as some longitudinal studies show that elevated emotional inertia can also precede or predict the later onset of MDD (Kuppens et al., 2012; van de Leemput et al., 2014).

Another possible reason could be related to the methodological aspects of the study. Specifically, it is possible that there was too little variability in the scores of items measuring NA to capture the differences in NA inertia, as almost all participants had more variability in items measuring positive emotions compared to negative. For some individuals, almost no variability was found in their ratings of the items capturing NA. As little variability is a problem in calculating autocorrelation, future studies could benefit from using longer EMA study durations or higher prompting frequencies that maximize the number of observations per participant. Moreover, although we excluded all days with less than 3 answered prompts per day (out of 5) from the analyses, these missing values could have influenced the calculation of the autocorrelation, especially when the missing prompts were either subsequent or intermittent.

Analogous to inertia, there might have been too little variability in the items of NA to adequately capture individual differences in participants’ NA differentiation indices. Furthermore, to reduce participant’s burden and to cover a wider range of emotional experience, two emotion words were grouped together in a single item (6 emotion items in total). This approach could have an effect on the calculation of emotion differentiation indices, and not accurately reflect the amount of emotion differentiation for each individual. Nonetheless, when comparing individuals with elevated depressive levels to those without (see Table 2), there was a trend for the individuals with elevated depressive symptoms to have less differentiated negative emotions (*p* < 0.05). Similar trend was also seen in individuals with elevated generalized anxiety symptoms. Hence, it would be useful to further look into the role of NA differentiation in future studies pertaining to depression and GAD, especially when bigger samples, longer study durations, and/or higher prompting frequencies could be implemented. Moreover, including a more diverse set of negative emotion items (*n* > 4) could be of help.

### 4.2 Joint models

The second major aim of this study was to explore which set of emotion dynamic indices best predict elevated depressive and GAD symptoms, when the mean levels of affect are controlled for, as suggested by Dejonckheere et al. (2019). In doing so, we found similar results for depression and generalized anxiety – namely, higher NA instability significantly increased the odds of having elevated depressive and GAD symptoms, even when PA and NA intensity were controlled for. What is more, the odds ratios for NA instability and NA intensity were of similar magnitude for both depression and generalized anxiety. The implications of these findings will be discussed below.

Importantly, the best set of predictors were identical for elevated depressive and GAD symptoms. This is surprising, as some specificity in affect dynamics has previously been found (e.g., Bosley et al., 2019). For instance, inertia appears to be primarily associated with depressive symptoms, whereas factors unique to anxiety disorders are generally not found. On one hand, these results could point toward the methodological limitations of the current study, as variability in emotion items might have been insufficient to capture individual differences in inertia and emotion differentiation. On the other hand, depressive and anxiety disorders are highly comorbid (Hirschfeld, 2001; Lamers et al., 2011), and thus, some similarities in the models could be expected. Taken together, our results point toward the role of emotion dysregulation in precipitating and/or maintaining depressive and GAD symptoms. As NA instability has previously been associated with various deficits in the emotion generative and regulatory processes (Kuppens and Verduyn, 2015; Trull et al., 2015), it is warranted more research in the context of both disorders.

In the final models, PA intensity also predicted elevated depressive and GAD symptoms, albeit to a lesser degree than other predictors, when comparing the ORs. Although PA intensity was not a significant predictor of elevated GAD symptoms in the final model, its contribution to generalized anxiety was almost identical to that of depression, when looking at the ORs and confidence intervals for the respective models (OR = 0.51, 99% CI 0.27–0.98 for depression, OR = 0.52, 99% CI 0.25–1.10 for generalized anxiety). What is more, PA intensity was a significant predictor of GAD symptoms in the final model when the relationship was modeled linearly (see Supplementary Table S3). In line with these findings, multiple studies have strongly linked PA dysregulation to depressive disorders (for review, see Gilbert, 2012). Moreover, some studies also demonstrate the link between PA dysregulation and various anxiety disorders (Kashdan, 2007; Kendall et al., 2015), even when the role of depressive mood or NA is controlled for (Eisner et al., 2009). Thus, although the contribution of NA dysregulation appears to be more important in depressive and anxiety disorders, the role of PA intensity still appears to be important alongside NA intensity and NA instability.

Lastly, the findings of a recent meta-analysis (Dejonckheere et al., 2019) suggest that studying more complex emotion dynamic indices might add little value in explaining psychological well-being, when affect intensity is taken into account. The results of our joint models do not appear to support this notion, as in both cases (for depression and generalized anxiety), model fit was improved when NA instability was added to models already containing mean NA and PA. In a similar vein, compared to NA intensity, the OR for NA instability was even slightly higher for depression, suggesting it has a bigger role in predicting elevated depression levels than mean NA. Nonetheless, it is worth mentioning that NA instability in predicting depressive symptoms was not significant alongside other variables in the final linear regression model (see Supplementary Table S3), suggesting that it is possible that the relationship between NA instability and depressive symptoms might not be linear.

Based on our results, then, the more subtle indices of emotional change, especially NA instability, should be further studied for both depressive and GAD symptoms. As instability of NA might be influenced by a multitude of factors, such as (a) not using proper emotion regulation strategies, (b) using too many different strategies, (c) being too reactive to the events in the inner/external environment, etc., the underlying mechanisms of NA instability should be further investigated, especially in relation to depressive and generalized anxiety symptoms. For instance, it is possible that depressed individuals might be passive and not engage in emotion regulation efforts, whereas anxious individuals might be overly sensitive to their threat-related and worrisome thoughts or bodily anxiety sensations. Both of these factors could be contributing to increased NA instability, but for successful intervention, precise mechanisms leading to NA instability should be disentangled.

### 4.3 Strengths, limitations and future directions

There are several strengths to this article. To start with, we included both men and women from various age groups, educational backgrounds, and income levels in our study. Moreover, unlike most studies in social sciences, we did not use a sample of university students, which should make our results more applicable to the general population. In fact, the mean age of our sample was 46 years (SD = 15 years). We also combined several different emotion dynamic indices in our analyses, which is a strength, as oftentimes, these indices are studied separately. Furthermore, we explored the role of positive emotion dysregulation in elevated depression and GAD symptoms, in addition to NA dysregulation.

In addition to the methodological issues discussed in the previous section, there are a few other limitations to the study. Importantly, participants with elevated depressive symptoms and participants with elevated generalized anxiety symptoms largely overlapped (i.e., 61%) in our sample. This is not surprising, as depressive and anxiety disorders are highly comorbid (Hirschfeld, 2001; Lamers et al., 2011). However, from the methodological perspective, the results of our models might be more applicable to depression than to generalized anxiety, as most of the sample with elevated generalized anxiety levels also showed elevated depressive symptoms, whereas only 11% of the sample had elevated GAD symptoms alone (compared to 26% of the sample having elevated depressive symptoms only). Therefore, future studies with larger sample sizes would benefit from differentiating between the groups. Furthermore, as those with a comorbid depression and generalized anxiety show greater psychiatric symptom severity and more functional impairment compared to those with only one disorder (Lamers et al., 2011; Kessler et al., 2015), comparing those patient groups on their emotion dynamics might help detect subtle nuances in affective processes that might inform future research as well as prevention and symptom management. In a similar vein, somatic symptoms of depression and GAD (e.g., insomnia, changes in appetite, mental fatigue, loss of energy, etc) should also be investigated, as they could have idiosyncratic associations with emotion dynamics.

Finally, as altered patterns of emotion dynamics are related to emotion dysregulation (Kuppens and Verduyn, 2015; Trull et al., 2015), the relationship between emotion dynamic indices and the use of various emotion regulation strategies is a fruitful avenue for future research. For example, Rónai and Polner (2021) recently demonstrated that greater NA instability and higher NA inertia mediated the relationship between rumination [i.e., a maladaptive emotion regulation strategy seen in depression and anxiety (Nolen-Hoeksema et al., 2008)], and NA intensity. In addition to dysfunctional emotion regulation strategies, similar studies could also help elucidate the adaptive emotion regulation strategies (i.e., reappraisal) that protect against the development or maintenance of altered affective dynamic patterns. Lastly, and as also proposed by Trull et al. (2015), it would be of use to study how the specific events in the environment (external or internal) trigger an emotional reaction and how these, in turn, relate to emotion dynamics.

### 4.4 Conclusion

In conclusion, we demonstrated that greater instability and intensity of negative affect and lower intensity of positive affect significantly increase the odds of having elevated depressive and GAD symptoms in a non-clinical sample of adults. These results point toward the need to further study the underlying emotion regulation processes, particularly those related to NA instability. Moreover, these results also demonstrate the utility of looking at the more nuanced emotion dynamic indices, beside the mean levels of affect. Taken together, the results highlight the role of NA fluctuations in depressive and anxiety disorders.

## Data availability statement

The datasets presented in this article are not readily available because of confidentiality restrictions. Requests to access the datasets should be directed to KK, kenn.konstabel@tai.ee.

## Ethics statement

The studies involving humans were approved by Research Ethics Committee of the National Institute for Health Development, Estonia. The studies were conducted in accordance with the local legislation and institutional requirements. The participants provided their written informed consent to participate in this study.

## Author contributions

HS: Conceptualization, Formal analysis, Methodology, Writing – original draft, Writing – review & editing. CM: Writing – review & editing, Methodology. MH: Methodology, Writing – review & editing. KK: Methodology, Writing – review & editing, Funding acquisition, Project administration, Supervision.

## Footnotes

^1^Of note, the terms ‘predict’ and ‘prediction’ refer to logistic regression models used in this article, as all analyses are cross-sectional.

^2^ www.formr.org

^3^ www.messenger.ee

^4^ https://osf.io/5fkpc/?view_only=ef4be4e84ca1438e9eacf3184639bd89

## References

[ref1] Aldao A. Gee D. G. De Los Reyes A. Seager I. (2016). Emotion regulation as a transdiagnostic factor in the development of internalizing and externalizing psychopathology: current and future directions. Dev. Psychopathol. 28, 927–946. doi: 10.1017/S0954579416000638, PMID: 27739387

[ref2] Aluoja A. Leinsalu M. Shlik J. Vasar V. Luuk K. (2004). Symptoms of depression in the Estonian population: prevalence, sociodemographic correlates and social adjustment. J. Affect. Disord. 78, 27–35. doi: 10.1016/S0165-0327(02)00179-9, PMID: 14672794

[ref3] Aluoja A. Shlik J. Vasar V. Luuk K. Leinsalu M. (1999). Development and psychometric properties of the emotional state questionnaire, a self-report questionnaire for depression and anxiety. Nord. J. Psychiatry 53, 443–449. doi: 10.1080/080394899427692

[ref4] Arslan R. C. Walther M. P. Tata C. S. (2020). Formr: a study framework allowing for automated feedback generation and complex longitudinal experience-sampling studies using R. Behav. Res. Methods 52, 376–387. doi: 10.3758/s13428-019-01236-y, PMID: 30937847 PMC7005096

[ref5] Barrett L. F. (2004). Feelings or words? Understanding the content in self-report ratings of experienced emotion. J. Pers. Soc. Psychol. 87, 266–281. doi: 10.1037/0022-3514.87.2.266, PMID: 15301632 PMC1351136

[ref6] Barrett L. F. Gross J. Christensen T. C. Benvenuto M. (2001). Knowing what you’re feeling and knowing what to do about it: mapping the relation between emotion differentiation and emotion regulation. Cognit. Emot. 15, 713–724. doi: 10.1080/02699930143000239

[ref7] Behrendt S. (2023). Im.beta: Add standardized regression coefficients to linear-model-objects (R Package Version 1.7-1). Available at: https://CRAN.R-project.org/package=lm.beta.

[ref8] Bosley H. G. Soyster P. D. Fisher A. J. (2019). Affect dynamics as predictors of symptom severity and treatment response in mood and anxiety disorders: evidence for specificity. J. Pers. Oriented Res. 5, 101–113. doi: 10.17505/jpor.2019.09, PMID: 33569146 PMC7842610

[ref9] Brose A. Schmiedek F. Koval P. Kuppens P. (2015). Emotional inertia contributes to depressive symptoms beyond perseverative thinking. Cognit. Emot. 29, 527–538. doi: 10.1080/02699931.2014.916252, PMID: 24820350

[ref10] Carstensen B. Plummer M. Laara E. Hills M. (2022). Epi: a package for statistical analysis in epidemiology. Available at: https://CRAN.R-project.org/package=Epi.

[ref11] Conrad A. Wilhelm F. H. Roth W. T. Spiegel D. Taylor C. B. (2008). Circadian affective, cardiopulmonary, and cortisol variability in depressed and nondepressed individuals at risk for cardiovascular disease. J. Psychiatr. Res. 42, 769–777. doi: 10.1016/j.jpsychires.2007.08.003, PMID: 17884093 PMC2478702

[ref12] Dejonckheere E. Mestdagh M. Houben M. Rutten I. Sels L. Kuppens P.. (2019). Complex affect dynamics add limited information to the prediction of psychological well-being. Nat. Hum. Behav. 3, 478–491. doi: 10.1038/s41562-019-0555-0, PMID: 30988484

[ref13] Demiralp E. Thompson R. J. Mata J. Jaeggi S. M. Buschkuehl M. Barrett L. F.. (2012). Feeling blue or turquoise? Emotional differentiation in major depressive disorder. Psychol. Sci. 23, 1410–1416. doi: 10.1177/0956797612444903, PMID: 23070307 PMC4004625

[ref14] Ebner-Priemer U. W. Eid M. Kleindienst N. Stabenow S. Trull T. J. (2009). Analytic strategies for understanding affective (in) stability and other dynamic processes in psychopathology. J. Abnorm. Psychol. 118, 195–202. doi: 10.1037/a0014868, PMID: 19222325

[ref15] Eisner L. R. Johnson S. L. Carver C. S. (2009). Positive affect regulation in anxiety disorders. J. Anxiety Disord. 23, 645–649. doi: 10.1016/j.janxdis.2009.02.001, PMID: 19278820 PMC2847490

[ref16] Erbas Y. Ceulemans E. Lee Pe M. Koval P. Kuppens P. (2014). Negative emotion differentiation: its personality and well-being correlates and a comparison of different assessment methods. Cognit. Emot. 28, 1196–1213. doi: 10.1080/02699931.2013.875890, PMID: 24410047

[ref17] Gilbert K. E. (2012). The neglected role of positive emotion in adolescent psychopathology. Clin. Psychol. Rev. 32, 467–481. doi: 10.1016/j.cpr.2012.05.005, PMID: 22710138

[ref18] Gratz K. L. Tull M. T. (2010). “Emotion regulation as a mechanism of change in acceptance -and mindfulness-based treatments” in Assessing mindfulness and acceptance processes in clients: illuminating the theory and practice of change. ed. Baer R. A. (Oakland, CA: Context Press/New Harbinger Publications)

[ref19] Hirschfeld R. M. (2001). The comorbidity of major depression and anxiety disorders: recognition and management in primary care. Prim. Care Companion J. Clin. Psychiatry 3, 244–254. doi: 10.4088/PCC.v03n0609, PMID: 15014592 PMC181193

[ref20] Hofmann S. G. Sawyer A. T. Fang A. Asnaani A. (2012). Emotion dysregulation model of mood and anxiety disorders. Depress. Anxiety 29, 409–416. doi: 10.1002/da.21888 22430982

[ref21] Houben M. Kuppens P. (2020). Emotion dynamics and the association with depressive features and borderline personality disorder traits: unique, specific, and prospective relationships. Clin. Psychol. Sci. 8, 226–239. doi: 10.1177/2167702619871962

[ref23] Jahng S. Wood P. K. Trull T. J. (2008). Analysis of affective instability in ecological momentary assessment: indices using successive difference and group comparison via multilevel modeling. Psychol. Methods 13, 354–375. doi: 10.1037/a0014173, PMID: 19071999

[ref24] Kashdan T. B. (2007). Social anxiety spectrum and diminished positive experiences: theoretical synthesis and meta-analysis. Clin. Psychol. Rev. 27, 348–365. doi: 10.1016/j.cpr.2006.12.003, PMID: 17222490

[ref25] Kendall A. D. Zinbarg R. E. Mineka S. Bobova L. Prenoveau J. M. Revelle W.. (2015). Prospective associations of low positive emotionality with first onsets of depressive and anxiety disorders: results from a 10-wave latent trait-state modeling study. J. Abnorm. Psychol. 124, 933–943. doi: 10.1037/abn0000105 26372005 PMC4658315

[ref26] Kessler R. C. Sampson N. A. Berglund P. Gruber M. J. Al-Hamzawi A. Andrade L.. (2015). Anxious and non-anxious major depressive disorder in the World Health Organization world mental health surveys. Epidemiol. Psychiatr. Sci. 24, 210–226. doi: 10.1017/S2045796015000189, PMID: 25720357 PMC5129607

[ref27] Koval P. Kuppens P. Allen N. B. Sheeber L. (2012). Getting stuck in depression: the roles of rumination and emotional inertia. Cognit. Emot. 26, 1412–1427. doi: 10.1080/02699931.2012.667392 22671768

[ref28] Koval P. Pe M. L. Meers K. Kuppens P. (2013). Affect dynamics in relation to depressive symptoms: variable, unstable or inert? Emotion 13, 1132–1141. doi: 10.1037/a0033579 23914765

[ref29] Koval P. Sütterlin S. Kuppens P. (2016). Emotional inertia is associated with lower well-being when controlling for differences in emotional context. Front. Psychol. 6:1997. doi: 10.3389/fpsyg.2015.01997 26779099 PMC4705270

[ref30] Kuppens P. Allen N. B. Sheeber L. B. (2010). Emotional inertia and psychological maladjustment. Psychol. Sci. 21, 984–991. doi: 10.1177/0956797610372634, PMID: 20501521 PMC2901421

[ref31] Kuppens P. Sheeber L. B. Yap M. B. Whittle S. Simmons J. G. Allen N. B. (2012). Emotional inertia prospectively predicts the onset of depressive disorder in adolescence. Emotion 12, 283–289. doi: 10.1037/a0025046, PMID: 21988744

[ref32] Kuppens P. Verduyn P. (2015). Looking at emotion regulation through the window of emotion dynamics. Psychol. Inq. 26, 72–79. doi: 10.1080/1047840X.2015.960505

[ref33] Laidra K. Reile R. Havik M. Leinsalu M. Murd C. Tulviste J.. (2023). Estonian National Mental Health Study: design and methods for a registry-linked longitudinal survey. Brain Behav. 13:e3106. doi: 10.1002/brb3.3106 37278143 PMC10454261

[ref34] Lamers F. van Oppen P. Comijs H. C. Smit J. H. Spinhoven P. van Balkom A. J.. (2011). Comorbidity patterns of anxiety and depressive disorders in a large cohort study: the Netherlands study of depression and anxiety (NESDA). J. Clin. Psychiatry 72, 341–348. doi: 10.4088/JCP.10m06176blu 21294994

[ref35] Mata J. Thompson R. J. Jaeggi S. M. Buschkuehl M. Jonides J. Gotlib I. H. (2012). Walk on the bright side: physical activity and affect in major depressive disorder. J. Abnorm. Psychol. 121, 297–308. doi: 10.1037/a0023533, PMID: 21553939 PMC3982878

[ref36] Minaeva O. George S. V. Kuranova A. Jacobs N. Thiery E. Derom C.. (2021). Overnight affective dynamics and sleep characteristics as predictors of depression and its development in women. Sleep 44:zsab129. doi: 10.1093/sleep/zsab129, PMID: 34013334 PMC8503829

[ref37] Nolen-Hoeksema S. Wisco B. E. Lyubomirsky S. (2008). Rethinking rumination. Perspect. Psychol. Sci. 3, 400–424. doi: 10.1111/j.1745-6924.2008.00088.x, PMID: 26158958

[ref38] Nowok B. Raab G. M. Dibben C. (2016). Synthpop: bespoke creation of synthetic data in R. J. Stat. Softw. 74, 1–26. doi: 10.18637/jss.v074.i11

[ref39] Ööpik P. Aluoja A. Kalda R. Maaroos H. I. (2006). Screening for depression in primary care. Fam. Pract. 23, 693–698. doi: 10.1093/fampra/cml052 17035287

[ref40] Peeters F. Berkhof J. Delespaul P. Rottenberg J. Nicolson N. A. (2006). Diurnal mood variation in major depressive disorder. Emotion 6, 383–391. doi: 10.1037/1528-3542.6.3.383 16938080

[ref41] Pfaltz M. C. Michael T. Grossman P. Margraf J. Wilhelm F. H. (2010). Instability of physical anxiety symptoms in daily life of patients with panic disorder and patients with posttraumatic stress disorder. J. Anxiety Disord. 24, 792–798. doi: 10.1016/j.janxdis.2010.06.001 20580527

[ref42] R Core Team (2021). R: a language and environment for statistical computing. R Foundation for Statistical Computing, Vienna, Austria.

[ref43] Revelle W. (2023). Psych: procedures for psychological, psychometric, and personality research. Northwestern University, Evanston, Illinois.

[ref44] Rónai L. Polner B. (2021). Getting the blues: negative affect dynamics mediate the within-person association of maladaptive emotion regulation and depression. Available at: https://osf.io/preprints/psyarxiv/he53c

[ref45] Schoevers R. A. Van Borkulo C. D. Lamers F. Servaas M. N. Bastiaansen J. A. Beekman A. T. F.. (2021). Affect fluctuations examined with ecological momentary assessment in patients with current or remitted depression and anxiety disorders. Psychol. Med. 51, 1906–1915. doi: 10.1017/S0033291720000689, PMID: 32234092 PMC8381239

[ref46] Shiffman S. Stone A. A. Hufford M. R. (2008). Ecological momentary assessment. Annu. Rev. Clin. Psychol. 4, 1–32. doi: 10.1146/annurev.clinpsy.3.022806.091415 18509902

[ref47] Sloan E. Hall K. Moulding R. Bryce S. Mildred H. Staiger P. K. (2017). Emotion regulation as a transdiagnostic treatment construct across anxiety, depression, substance, eating and borderline personality disorders: a systematic review. Clin. Psychol. Rev. 57, 141–163. doi: 10.1016/j.cpr.2017.09.002, PMID: 28941927

[ref48] Stanton K. Watson D. (2014). Positive and negative affective dysfunction in psychopathology. Soc. Personal. Psychol. Compass 8, 555–567. doi: 10.1111/spc3.12132

[ref49] Stickley A. Leinsalu M. (2018). Childhood hunger and depressive symptoms in adulthood: findings from a population-based study. J. Affect. Disord. 226, 332–338. doi: 10.1016/j.jad.2017.09.013 29031183

[ref50] Thompson R. J. Mata J. Jaeggi S. M. Buschkuehl M. Jonides J. Gotlib I. H. (2012). The everyday emotional experience of adults with major depressive disorder: examining emotional instability, inertia, and reactivity. J. Abnorm. Psychol. 121, 819–829. doi: 10.1037/a0027978, PMID: 22708886 PMC3624976

[ref51] Trull T. J. Lane S. P. Koval P. Ebner-Priemer U. W. (2015). Affective dynamics in psychopathology. Emot. Rev. 7, 355–361. doi: 10.1177/1754073915590617, PMID: 27617032 PMC5016030

[ref52] van de Leemput I. A. Wichers M. Cramer A. O. Borsboom D. Tuerlinckx F. Kuppens P.. (2014). Critical slowing down as early warning for the onset and termination of depression. Proc. Natl. Acad. Sci. 111, 87–92. doi: 10.1073/pnas.1312114110, PMID: 24324144 PMC3890822

