# Supplementary material for: Negative affect instability predicts elevated depressive and generalized anxiety disorder symptoms even when negative affect intensity is controlled for: an ecological momentary assessment study
Source: Front Psychol. 2024 Apr 23;15:1371115. doi: 10.3389/fpsyg.2024.1371115 (PMC11074391; doi:10.3389/fpsyg.2024.1371115)
Supplement: Supplementary file 1 [file Data_Sheet_1.PDF]

Supplementary Material

Table 1. Ecological momentary assessment (EMA) questionnaire, adapted from Laidra et al. (2023).

	Item	Response options
	**At this moment, how strongly do you feel…** *Mark on each row how strongly do you currently experience one or more of these feelings*	
1	**joyful, excited**	Not at all ----- Very strongly (7-point scale)
2	**satisfied, relaxed**	Not at all ----- Very strongly (7-point scale)
3	**worried, anxious**	Not at all ----- Very strongly (7-point scale)
4	**sad, disappointed**	Not at all ----- Very strongly (7-point scale)
5	**irritated, angry**	Not at all ----- Very strongly (7-point scale)
6	**tense, stressed**	Not at all ----- Very strongly (7-point scale)
7	**tired, listless**	Not at all ----- Very strongly (7-point scale)
8	**What are Your current feelings related to?** *Choose all relevant options.*	a. An ongoing situation<br>b. An earlier situation<br>c. A situation in the future<br>d. Same situation I mentioned in the previous prompt<br>e. Work or studies<br>f. Spending leisure time<br>g. Close relationships<br>h. Other relationships<br>i. COVID-19<br>j. Celebrating something<br>k. Eating<br>l. Something else/ hard to say
9	**What have you tried to do to make yourself feel better?** *Mark all strategies you have used since the previous prompt.*	a. I haven't tried anything<br>b. I suppressed my feelings<br>c. I acted out my feelings<br>d. I solved problems causing these emotions<br>e. I distracted myself with something to take my mind off my troubles<br>f. I thought how things are not as bad as they initially seemed<br>g. I thought about how bad things can be good for something<br>h. I accepted that things are as they are<br>i. I meditated or did relaxation exercises<br>j. I was physically active or worked out<br>k. I smoked<br>l. I consumed alcohol or other drugs<br>m. I ate or snacked on something<br>n. I ate more than I would have liked<br>o. I shought emotional support from my loved ones<br>p. I sought professional help or support<br>r. I did something else: _________
10	**Have these techniques made you feel any better?**	Not at all ----- A lot better (7-point scale)
11*	**How well did you sleep last night?**	Very unwell ----- Very well (7-point scale)
12*	**What time did you go to bed last night?**	[drop-down menu with 15-min intervals**]
13*	**What time did you wake up this morning?**	[drop-down menu with 15-min intervals**]
14*	**How many minutes did you engage in moderate to high physical activity yesterday?** *(f.e. practised sports, worked out or went for a walk so that it made you sweat or gasp lightly)*	Insert minutes ______
15*	**How many minutes did you engage in light physical activity yesterday?** *(f.e. slow-paced walk or cycling/bike ride)*	Insert minutes ______
16*	**Did you consume any alcoholic beverages yesterday?**	Yes / No
17*	**How do you assess your health at the moment?**	Very bad ------ Very good (7-point scale)

Note: * Items included only in the first prompt of each day.

References

Laidra, K., Reile, R., Havik, M., Leinsalu, M., Murd, C., Tulviste, J., ... & Konstabel, K. (2023). Estonian National Mental Health Study: Design and methods for a registry-linked longitudinal survey. *Brain and Behavior*, e3106.

Table 2. Standardized regression coefficients for individual emotion dynamic indices predicting elevated depressive and generalized anxiety disorder symptoms.

	**Depressive symptoms**				**Generalized anxiety disorder symptoms**			
**Variable**	***B***	***SE***	***t***	***p***	***B***	***SE***	***t***	***p***
PA intensity	-.29	.37	-4.77	**<.001**	-.29	.29	-4.75	**<.001**
NA intensity	.59	.43	11.54	**<.001**	.56	.34	10.51	**<.001**
PA instability	.18	1.02	2.76	**.0063**	.15	.79	2.28	.0239
NA instability	.46	1.06	8.13	**<.001**	.52	.79	9.54	**<.001**
PA inertia	-.05	1.12	-.81	.4168	-.04	.87	-.64	.5213
NA inertia	.04	1.10	.62	.539	.07	.85	1.03	.3030
PA differentiation	-.04	.88	-.65	.5174	.03	.69	.50	.6208
NA differentiation	.10	1.46	1.40	.165	.17	1.10	2.50	.0132

Note: *B* – standardized regression coefficient, NA – negative affect, PA – positive affect, *SE* – standard error. All predictors were adjusted for age and gender.

Table 3. Results of the initial and final linear regression models predicting elevated depressive and generalized anxiety disorder symptoms.

	**Depressive symptoms (n = 218)**							
	**Initial model**				**Final model**			
**Variable**	***B***	***SE***	***t***	***p***	***B***	***SE***	***t***	***p***
(Intercept)	9.42	1.97	4.79	**<.001**	8.44	2.03	4.17	**<.001**
Gender (female)	.05	.78	1.0	.3183	.05	.78	1.08	.2819
Age	-.22	.02	- 4.38	**<.001**	-.22	.02	-4.34	**<.001**
PA intensity	-.13	.32	-2.41	.0168	-.13	.31	-2.47	.0145
NA intensity	.55	.44	10.38	**<.001**	.47	.58	6.67	**<.001**
NA instability					.13	1.26	1.88	**.0617**
	**Generalized anxiety disorder symptoms (n = 219)**							
	**Initial model**				**Final model**			
**Variable**	***B***	***SE***	***t***	***p***	***B***	***SE***	***t***	***p***
(Intercept)	7.76	1.58	4.92	**<.001**	6.15	1.57	3.91	**<.001**
Gender (female)	.08	.63	1.50	.1344	.09	.61	1.71	.0886
Age	-.20	.02	-3.88	**<.001**	-.20	.02	-3.90	**<.001**
PA intensity	-.14	.25	-2.55	.0114	-.15	.25	-2.75	**.0065**
NA intensity	.52	.35	9.38	**<.001**	.33	.45	4.69	**<.001**
NA instability					.27	.98	4.03	**<.001**

Note: *B* – regression coefficient, NA – negative affect, PA – positive affect, *SE* – standard error.
